# Artificial Intelligence in the Fight Against Antimicrobial Resistance: Applications in Diagnosis, Susceptibility Prediction, Precision Dosing, and Antibiotic Discovery

**DOI:** 10.3390/antibiotics15100983

**Published:** 2026-10-06

**Authors:** Susanna Esposito, Valentina Fainardi, Gaia Giorgia Arnesano, Alberto Argentiero, Nicola Principi

**Affiliations:** 1 Pediatric Clinic, Department of Medicine and Surgery, University of Parma, 43126 Parma, Italy; valentina.fainardi@unipr.it (V.F.); gaiagiorgia.arnesano@unipr.it (G.G.A.); alberto.argentiero@unipr.it (A.A.); 2 Department of Pathophysiology and Transplantation, University of Milan, 20122 Milan, Italy; nicola.principi@unimi.it

**Keywords:** antimicrobial resistance, artificial intelligence, antimicrobial stewardship, machine learning, precision dosing, antibiotic discovery

## Abstract

Background: Antimicrobial resistance (AMR) is a major global health threat, while conventional diagnostic, stewardship, dosing, and drug-development strategies remain limited by diagnostic delays, population-level decision-making, and a shrinking antibiotic pipeline. Artificial intelligence (AI) may help address these gaps by integrating complex clinical, microbiological, genomic, pharmacological, and chemical data. Methods: We conducted a narrative review of English-language publications issued between 1 January 2010 and 27 August 2026. PubMed/MEDLINE, Scopus, and Web of Science were searched using a prespecified combination of terms related to AI, machine learning, AMR, diagnosis, susceptibility prediction, antimicrobial stewardship, precision dosing, pharmacokinetics, pharmacodynamics, and antibiotic discovery. A separate structured search of ClinicalTrials.gov, the World Health Organization International Clinical Trials Registry Platform, and ISRCTN was performed on 27 August 2026 to identify prospective interventional studies. Results: AI-based systems demonstrated promising performance in differentiating bacterial from viral infections, predicting bacteremia and sepsis, enhancing matrix-assisted laser desorption/ionization time-of-flight interpretation, estimating antimicrobial susceptibility, supporting individualized empirical therapy, optimizing antibiotic dosing, and accelerating compound discovery. However, external and temporal validation frequently produced substantially poorer performance than internal validation. In representative resistance-prediction studies, AUROC declined from 0.94 internally to 0.55 during temporally independent external validation and by 0.10–0.25 over 18 months. Moreover, the structured registry search identified five prospective interventional studies of AI-based applications and one additional randomized study of conventional model-informed precision dosing. Conclusions: Current evidence demonstrates that AI can generate technically promising predictions, but it does not yet establish consistent clinical or economic benefit. Discrimination alone is insufficient to demonstrate clinical usefulness. Prospective multicenter trials, clinically meaningful outcome measures, external validation, continuous monitoring for model drift, and formal implementation and cost-effectiveness evaluations are required before widespread adoption.

## 1. Introduction

Antimicrobial resistance (AMR) is among the most serious threats to global health. In 2021, bacterial AMR was estimated to have directly caused 1.14 million deaths and contributed to 4.71 million deaths worldwide. Without effective interventions, this burden is expected to increase substantially, with more than 39 million AMR-related deaths projected between 2025 and 2050 [1]. Although the greatest burden is borne by low- and middle-income countries, AMR also represents a major and growing challenge in Europe and North America, where multidrug-resistant organisms, including extended-spectrum β-lactamase (ESBL)-producing and carbapenem-resistant *Enterobacterales*, methicillin-resistant *Staphylococcus aureus* (MRSA), and multidrug-resistant *Pseudomonas aeruginosa*, are increasingly reported.

The effects of AMR are unevenly distributed across age groups. Children younger than 5 years, particularly neonates, remain highly vulnerable, and approximately 20% of Gram-negative bloodstream isolates in pediatric patients are resistant to third-generation cephalosporins. At the opposite end of the age spectrum, adults aged ≥70 years constitute the fastest-growing group at risk of AMR-related mortality. Beyond its direct impact on survival, AMR is associated with treatment failure, recurrent infection, sepsis, organ dysfunction, prolonged hospitalization, and increased admission to intensive care. It also imposes a substantial economic burden through higher healthcare expenditures, longer hospital stays, the use of costly second-line therapies, and productivity losses. Moreover, AMR threatens the safety and effectiveness of modern medical procedures that depend on reliable antimicrobial prophylaxis and treatment, including organ transplantation, cancer chemotherapy, major surgery, and neonatal intensive care.

Current strategies to address AMR rely primarily on the development of new antibiotics and the implementation of antimicrobial stewardship programs (ASPs), supported by infection prevention, surveillance, vaccination, and broader public health interventions. ASPs seek to optimize antibiotic use through guideline-based prescribing, dose adjustment, microbiology-guided de-escalation, and the avoidance of unnecessary broad-spectrum therapy. Although these programs have demonstrated substantial value [2,3], their effectiveness is constrained by several important limitations [4,5]. Conventional microbiological cultures and antimicrobial susceptibility testing generally require 24–72 h, compelling clinicians to initiate empirical treatment before the causative pathogen and its resistance profile are known. In addition, treatment recommendations are often based on population-level antibiograms rather than individualized patient characteristics, while many stewardship interventions remain labor-intensive, retrospective, and difficult to implement consistently. At the same time, antibiotic development has slowed markedly despite the increasing prevalence of multidrug-resistant infections, resulting in a progressively limited antimicrobial pipeline.

Artificial intelligence (AI) offers new opportunities to address several of these challenges by integrating clinical, microbiological, pharmacological, genomic, and epidemiological data to support timely and individualized decision-making. AI-based systems may improve diagnostic accuracy, distinguish bacterial from viral infections, predict antimicrobial resistance and susceptibility before conventional laboratory results become available, optimize antibiotic dosing, and guide the selection of empirical therapy. AI may also accelerate antibiotic discovery by enabling the rapid screening of large chemical libraries, identifying novel antimicrobial candidates, and supporting molecular optimization and drug-development processes.

By facilitating more personalized antimicrobial stewardship and expanding the capacity to identify new therapeutic candidates, AI could strengthen existing strategies to reduce the clinical and public health burden of AMR. However, high performance during model development does not necessarily indicate that an algorithm will remain accurate across institutions or periods, change antimicrobial prescribing, improve patient outcomes, or be economically sustainable [6,7,8]. This narrative review therefore aims not only to summarize the most advanced applications of AI in diagnosis, resistance prediction, precision dosing, and antibiotic discovery, but also to examine the gap between technical performance and demonstrated clinical benefit. Particular attention is given to external validity, temporal model drift, clinically meaningful performance measures, prospective interventional evidence, implementation requirements, and economic considerations.

## 2. Methods

This narrative review examined the applications of AI to AMR, focusing on four prespecified domains: infection diagnosis, antimicrobial-resistance or -susceptibility prediction and stewardship, model-informed precision dosing, and antibiotic discovery. The literature search and study selection were structured to improve transparency and reproducibility, although the review was not designed as a systematic review or meta-analysis.

### 2.1. Information Sources and Literature-Search Strategy

PubMed/MEDLINE, Scopus, and Web of Science were searched for English-language publications issued between 1 January 2010 and 27 August 2026. The final search was performed on 27 August 2026. The search combined terms describing AI methods with terms related to AMR and the four review domains. The PubMed/MEDLINE search strategy was: (“artificial intelligence”[Title/Abstract] OR “machine learning”[Title/Abstract] OR “deep learning”[Title/Abstract] OR “neural network*”[Title/Abstract] OR “clinical decision support”[Title/Abstract] OR “digital twin*”[Title/Abstract]) AND (“antimicrobial resistance”[Title/Abstract] OR “antibiotic resistance”[Title/Abstract] OR “antimicrobial susceptibility”[Title/Abstract] OR “antibiotic stewardship”[Title/Abstract] OR “bacterial infection”[Title/Abstract] OR bacteremia[Title/Abstract] OR sepsis[Title/Abstract] OR “precision dosing”[Title/Abstract] OR “model-informed precision dosing”[Title/Abstract] OR pharmacokinetic*[Title/Abstract] OR pharmacodynamic*[Title/Abstract] OR “antibiotic discovery”[Title/Abstract] OR “antimicrobial discovery”[Title/Abstract] OR “antimicrobial peptide*”[Title/Abstract]).

The strategy was adapted to the syntax and indexing conventions of Scopus and Web of Science. In Scopus, the same terms were searched in the title, abstract, and keyword fields; in Web of Science, they were searched as topic terms. Searches were restricted to publications in English and to the specified date range. The reference lists of eligible publications and relevant reviews were also screened to identify additional studies. Technical reports, guidelines, and policy documents issued by the World Health Organization and other relevant international organizations were identified through targeted searches of their official websites.

### 2.2. Eligibility Criteria and Study Selection

Eligible publications included randomized and non-randomized interventional studies, prospective and retrospective observational studies, diagnostic and prognostic studies, external-validation studies, pharmacokinetic and pharmacodynamic investigations, and systematic reviews addressing an AI-based application in at least one of the four prespecified domains. Narrative reviews, international guidelines, and programmatic or technical reports were used to provide background or implementation context. Studies were eligible when an AI, machine-learning, deep-learning, or model-informed method was a substantive component of infection classification, resistance or susceptibility prediction, antimicrobial selection or stewardship, individualized antibiotic dosing, or antimicrobial discovery.

Publications were excluded if they did not address bacterial infection or antibacterial therapy; described only conventional statistical or computerized methods without an AI, machine-learning, or model-informed component; did not report information relevant to the review objectives; were conference abstracts without sufficient methodological or outcome data; or were not available in English. Duplicate records were removed before screening. Titles and abstracts were assessed for relevance, followed by full-text assessment of potentially eligible publications. Uncertainties regarding eligibility were resolved through discussion among the authors. Because this was a narrative review, the literature was selected to represent the principal applications, the strongest available clinical evidence, and important limitations rather than to support pooled effect estimates.

For each included study discussed in detail, the authors extracted the clinical or experimental setting, study design, population or dataset, AI approach, validation strategy, principal performance measures, and reported clinical, implementation, or discovery outcomes. Greater interpretative weight was assigned to prospective studies, multicenter investigations, external or temporal validations, randomized studies, international recommendations, and studies reporting clinically meaningful outcomes. No meta-analysis was undertaken because of substantial heterogeneity in populations, data sources, algorithms, validation methods, comparators, and outcome definitions. No formal risk-of-bias assessment was performed; this limitation was considered when interpreting the evidence.

### 2.3. Operational Definition and Classification of Artificial Intelligence Methods

For this review, AI was operationally defined as a computational approach in which a model learns patterns or decision functions from data to generate classifications, predictions, recommendations, or novel candidate structures. Machine learning was considered a subset of AI, and deep learning was considered a subset of machine learning based on multilayer neural networks. Eligible AI methods included supervised and unsupervised machine-learning algorithms, ensemble-learning methods, neural networks, deep-learning architectures, and generative models.

The underlying analytical platform or data source was not considered sufficient to classify an application as AI. Thus, MALDI-TOF mass spectrometry, whole-genome sequencing, therapeutic drug monitoring, and electronic clinical decision-support systems were classified as AI-based only when their outputs were analyzed or interpreted using a machine-learning or deep-learning model. Conventional spectral matching, fixed laboratory algorithms, rule-based alerts, and computerized decision-support systems without a learning component were not classified as AI.

Similarly, population pharmacokinetic models, conventional Bayesian forecasting, nonparametric pharmacokinetic platforms, and model-informed precision dosing were not considered AI merely because they generated individualized dose recommendations. They were classified as AI-based only when machine learning was used for exposure prediction, model selection, model averaging, dose optimization, or another substantive component of the dosing procedure. Conventional pharmacokinetic and Bayesian approaches were retained only when they served as comparators or provided relevant context for evaluating AI-assisted dosing. Following application of this operational definition, the included studies were reassessed and categorized as AI or machine-learning applications, hybrid approaches combining AI with conventional models, or adjacent conventional computational methods.

### 2.4. Extraction and Interpretation of Model Performance and Clinical Outcomes

For each study discussed in detail, we considered the study design, clinical setting, sample size, outcome prevalence, handling of missing data, model-development procedure, validation strategy, comparator, and reported performance measures. Performance measures were extracted as reported by the original investigators; no missing measures were calculated from incomplete published data. Measures that were not reported by the primary study were recorded as not reported and were considered when evaluating the completeness and clinical interpretability of the evidence.

Model performance was evaluated across three distinct levels: technical performance, potential clinical utility, and demonstrated clinical benefit. Technical performance included discrimination, accuracy, sensitivity, specificity, predictive values, calibration, prediction error, and performance during internal, external, or temporal validation. Outcome prevalence was considered essential for interpreting predictive values. Particular attention was given to whether decision thresholds were prespecified or selected after model development, whether missing data were described and appropriately handled, and whether measures were taken to limit overfitting, including cross-validation, independent test sets, external validation, temporal validation, regularization, and appropriate separation of training and validation data.

For antimicrobial-susceptibility prediction, overall accuracy and AUROC were not considered sufficient to determine clinical reliability. When reported, we preferentially considered categorical agreement and the rates of very major errors, major errors, and minor errors. A very major error, in which a resistant isolate is incorrectly classified as susceptible, was regarded as particularly important because it may lead to ineffective treatment. Major errors, in which a susceptible isolate is classified as resistant, may unnecessarily restrict treatment options or promote the use of broader-spectrum agents. The definitions and denominators reported by the original studies were retained because they were not uniform across all publications.

Potential clinical utility was defined as the capacity of a model to influence a clinically relevant decision, such as whether to obtain a blood culture, initiate or withhold an antibiotic, select empirical therapy, adjust a dose, or prioritize a candidate compound. Demonstrated clinical benefit required prospective evidence that use of the model improved patient- or health-system outcomes, including treatment failure, toxicity, time to effective therapy, antimicrobial exposure, length of hospitalization, mortality, emergence of resistance, or cost-effectiveness.

Reported effects were classified according to their level of evidence as: (1) simulated or model-estimated effects; (2) retrospectively estimated effects based on historical data; (3) prospectively observed effects in routine or interventional clinical use; or (4) effects demonstrated in a randomized controlled study. Improvements in predictions, simulated decisions, or pharmacokinetic target attainment were not interpreted as demonstrated clinical benefits unless corresponding effects on patient care or clinical outcomes were prospectively observed.

Reporting of outcome prevalence, missing-data procedures, calibration, decision thresholds, and susceptibility-classification errors was inconsistent across the included studies. Consequently, direct comparisons between models were not always possible. The absence of these measures was treated as a limitation rather than interpreted as evidence of satisfactory performance. Because this was a narrative review and individual-patient data were unavailable, we did not independently recalculate calibration measures, predictive values, or categorical error rates.

Because the included studies varied substantially in design and maturity, their findings were interpreted according to their validation and evidence stage. Development studies with internal validation were distinguished from geographic or temporal external-validation studies, retrospective clinical-impact estimations, prospective observational evaluations, and randomized interventional studies. These categories were described in the accompanying narrative for each study. The evidence tables were retained as concise summaries of the principal applications and reported findings, whereas study design, validation stage, and limitations were addressed in the corresponding text.

### 2.5. Structured Search for Prospective Interventional Studies

Because the number of prospective interventional studies was reported quantitatively, a separate structured search was conducted in ClinicalTrials.gov, the World Health Organization International Clinical Trials Registry Platform, and ISRCTN. All three registries were searched on 27 August 2026, without imposing the earlier 31 March 2026 eligibility cutoff.

Searches combined the terms “artificial intelligence,” “machine learning,” “deep learning,” “clinical decision support,” or “model-informed precision dosing” with “antibiotic,” “antimicrobial,” “antimicrobial resistance,” “antibiotic stewardship,” “bacterial infection,” “susceptibility,” “precision dosing,” “vancomycin,” or “antibiotic discovery.” Searches were performed using individual combinations of these terms when registry interfaces did not support the complete Boolean strategy.

For the primary analysis of AI-based prospective evidence, eligible records were prospective interventional studies in which a machine-learning or other AI component contributed directly to infection diagnosis, antimicrobial selection, resistance or susceptibility prediction, antimicrobial stewardship, individualized dosing, or antibiotic discovery. Prospective studies of conventional Bayesian forecasting or model-informed precision dosing without a machine-learning component were identified separately as adjacent pharmacometric evidence and were not counted as AI interventions. The registry search was intended as a structured, targeted search of the prespecified registries rather than as proof that no other potentially relevant study existed outside these sources.

## 3. Artificial Intelligence-Based Diagnostic Systems

### 3.1. Artificial Intelligence for Differentiating Bacterial and Viral Infections

One of the most relevant applications of AI in antimicrobial stewardship is the identification of patients with suspected infectious diseases who are likely to have a bacterial infection requiring antibiotic treatment and, conversely, those whose illness is more likely to be viral and therefore unlikely to benefit from antimicrobial therapy. This distinction remains challenging in routine clinical practice because bacterial and viral infections often present with overlapping signs and symptoms, particularly in respiratory tract infections, febrile illnesses, and pediatric emergency settings. Although biomarkers such as C-reactive protein (CRP) and procalcitonin (PCT) are widely used to support clinical decision-making, their diagnostic performance is often suboptimal, especially when values fall within intermediate ranges. In these situations, diagnostic uncertainty frequently leads to empirical antibiotic prescribing [9,10]. AI-based clinical decision-support systems may help overcome these limitations by integrating demographic, clinical, laboratory, and molecular data to generate individualized estimates of infection etiology.

A relatively straightforward example is the machine-learning model developed by Gunčar et al. [11], which incorporated 16 routine laboratory parameters together with CRP, age, and sex in a cohort of more than 44,000 patients. The model achieved an area under the receiver operating characteristic curve (AUROC) of 0.905, with an accuracy of 82.2%, a sensitivity of 79.7%, and a specificity of 84.5% for distinguishing bacterial from viral infections, thereby outperforming CRP-based diagnostic approaches. The greatest benefit was observed in patients with CRP concentrations between 10 and 40 mg/L, a clinically relevant diagnostic “gray zone” in which antibiotic overprescribing is particularly common.

More advanced approaches have focused on host transcriptional responses rather than conventional laboratory variables. Mayhew et al. developed a neural-network classifier based on the expression of 29 host mRNAs selected from large transcriptomic datasets [12]. The model was trained and validated across multiple independent cohorts and achieved AUROCs of 0.92 (95% CI, 0.90–0.93) for both bacterial-versus-other and viral-versus-other classifications, demonstrating that infection etiology can be accurately predicted using a relatively small host gene-expression signature.

Similarly, Li et al. integrated transcriptomic data from 2680 patients across 16 cohorts to develop the bacterial–viral–noninfected Gene Pair Signature (bvnGPS), a multiclass neural-network model [13]. The classifier achieved AUROCs of 0.953 (95% CI, 0.948–0.958) and 0.956 (95% CI, 0.951–0.961) for bacterial and viral infections, respectively, with corresponding external-validation AUROCs of 0.988 and 0.994.

Further advances were reported by Shen et al. [14], who developed InfectDiagno, an ensemble machine-learning algorithm based on host gene-expression profiles. After selecting 100 informative genes, the model achieved an AUROC of 0.95 (95% CI, 0.93–0.97) for bacterial-versus-viral discrimination. Sensitivities were 93.1% and 87.2%, and specificities were 96.3% and 92.9%, for bacterial and viral infections, respectively. In a prospective validation cohort of 517 patients, approximately 95% of samples were correctly classified.

Collectively, these findings suggest that AI-assisted interpretation of routine laboratory data and host transcriptional responses may substantially improve the differentiation of bacterial and viral infections. Such systems could support more judicious antibiotic prescribing, particularly when microbiological confirmation is unavailable or delayed.

### 3.2. Artificial Intelligence for Predicting Bacteremia and Invasive Bacterial Infection

Beyond classifying infection etiology, AI has increasingly been used to identify patients at risk of bacteremia or other invasive bacterial infections before microbiological confirmation becomes available. Kaal et al. [15] developed and externally validated a prediction model incorporating routine clinical variables and procalcitonin concentrations in emergency-department patients with suspected bloodstream infection. During external validation, the model achieved an AUROC of 0.87. At the evaluated decision threshold, a retrospective model-based analysis estimated that blood-culture utilization could have been reduced by 29% while 1.1% of bacteremia cases would have been missed. These findings represent an estimated effect on clinical decisions rather than a prospectively observed reduction in blood-culture use.

Similarly, Choi et al. [16] developed and externally validated a Bayesian neural network-based model for bacteremia prediction using both structured and unstructured emergency department data. The model demonstrated good external-validation performance, with an AUROC of 0.738 and a sensitivity of 92.7%. It was subsequently evaluated as a clinical decision-support tool by providing physicians with AI-generated risk estimates and information regarding predictive uncertainty. Access to these estimates significantly improved physicians’ diagnostic performance, increasing the AUROC from 0.639 to 0.703 (*p* < 0.001). The greatest benefit was observed when clinicians had low confidence in their initial assessments and the AI model generated predictions with low uncertainty.

These findings indicate that uncertainty-aware AI systems may improve bacteremia risk stratification and support clinical decision-making, particularly in diagnostically challenging cases. Although primarily designed to enhance diagnostic stewardship, these approaches may also contribute to antimicrobial stewardship by reducing unnecessary investigations and empirical antibiotic exposure in patients with a very low probability of invasive bacterial infection.

### 3.3. Artificial Intelligence for Early Prediction of Sepsis, Clinical Deterioration, and Antibiotic Need

Related AI applications have focused on identifying patients at risk of severe bacterial disease, sepsis, or clinical deterioration. Using data from 136,478 intensive care unit admissions across the United States, the Netherlands, and Switzerland, Moor et al. [17] developed a deep-learning system for early sepsis prediction. The model achieved an internal-validation AUROC of 0.846 and identified 80% of patients who developed sepsis a median of 3.7 h before clinical onset.

In the pediatric setting, Velez et al. [18] developed a two-stage machine-learning framework using electronic health record (EHR) data from 5706 non-immunocompromised children treated in six pediatric emergency departments. The first model predicted clinical deterioration among children initially managed without antibiotics, whereas the second estimated the probability of bacteremia among children receiving empirical antimicrobial therapy. Both models achieved negative predictive values greater than 96%, indicating a strong ability to identify low-risk patients who were unlikely to develop bacteremia or experience clinical deterioration. However, the study did not prospectively evaluate an intervention in which antibiotics were withheld on the basis of model predictions. The findings therefore establish technical performance and potential clinical utility, but not the safety of AI-guided withholding of antibiotics in routine practice.

These findings suggest that AI-based risk-stratification tools may support earlier recognition of severe infection while also identifying patients in whom antibiotics can potentially be withheld or discontinued safely. Nevertheless, prospective studies are needed to determine whether these models improve clinical outcomes and reduce unnecessary antimicrobial exposure in routine practice.

### 3.4. Artificial Intelligence-Enhanced Microbiological Diagnostics Using Matrix-Assisted Laser Desorption/Ionization Time-of-Flight Mass Spectrometry

AI is increasingly being integrated with matrix-assisted laser desorption/ionization time-of-flight mass spectrometry (MALDI-TOF MS) to enhance microbiological diagnosis. Although MALDI-TOF MS is already widely used for species identification in clinical microbiology laboratories, conventional workflows exploit only a limited proportion of the information contained within the generated spectra. Machine-learning and deep-learning algorithms can analyze complete spectral profiles and identify complex patterns that may not be detected through traditional database-matching approaches.

Recent studies have shown that AI-based analysis of MALDI-TOF spectra can accurately classify bacterial species, distinguish closely related organisms, and, in some cases, infer antimicrobial-resistance phenotypes directly from routinely generated data. One of the largest examples is the ANTIBIOTIC platform developed by Wang et al. [19], which was trained using the DRIAMS database and 89,026 MALDI-TOF spectra obtained during routine clinical practice. The platform combined 26 machine-learning models for bacterial identification with 248 deep-learning models for antimicrobial-resistance prediction. For species identification, the system achieved a median area under the curve (AUC) of 0.99 during internal validation and 0.96 during temporally independent external validation.

These results indicate that AI can extract clinically useful information from routinely generated MALDI-TOF spectra and may provide highly accurate bacterial identification without requiring additional laboratory procedures or diagnostic platforms. The possibility of simultaneously predicting species identity and resistance phenotypes could further shorten the interval between specimen collection and targeted antimicrobial treatment.

Despite these promising findings, the clinical implementation of AI-based diagnostic systems remains limited. Many models have been developed and validated in selected populations, raising concerns regarding external generalizability. Additional challenges include temporal performance degradation, geographic variation in pathogen and resistance patterns, heterogeneity in data acquisition and preprocessing, limited interpretability, and dependence on high-quality local datasets. Moreover, prospective interventional studies demonstrating that these technologies improve patient outcomes, reduce antibiotic use, or enhance antimicrobial stewardship remain scarce. Therefore, broader implementation will require external and prospective validation, regular model recalibration, transparent reporting, and integration into existing clinical and laboratory workflows.

The principal applications, data sources, performance measures, and potential contributions of AI-based diagnostic systems to antimicrobial stewardship are summarized in Table 1.

Despite the generally promising discrimination reported for AI-assisted diagnosis, AUROC and accuracy do not establish that a diagnostic model will improve antimicrobial use or patient outcomes. Clinical usefulness depends on sensitivity and negative predictive value at thresholds at which antibiotics can safely be withheld, specificity and positive predictive value at thresholds used to initiate or escalate treatment, calibration across clinically relevant risk groups, and the consequences of false-negative and false-positive classifications. It also depends on whether results are available early enough to influence treatment and whether clinicians act on the recommendations.

Most studies reviewed in this section were retrospective or evaluated archived samples and therefore could not determine whether implementation reduced antibiotic initiation, shortened treatment, accelerated appropriate therapy, or improved clinical outcomes. The reported technical performance should consequently be interpreted as evidence of diagnostic potential rather than evidence of clinical effectiveness. Prospective interventional studies incorporating antibiotic-use and patient-safety outcomes remain necessary.

## 4. Artificial Intelligence for Antimicrobial Susceptibility Prediction

Even after a bacterial pathogen has been identified, effective antibiotic treatment depends on the timely determination of antimicrobial susceptibility. Conventional antimicrobial susceptibility testing (AST) requires pathogen isolation followed by phenotypic testing, and definitive results generally become available 24–72 h after culture positivity. Consequently, patients with severe infections are frequently treated empirically with broad-spectrum antibiotics. Although this approach may be lifesaving, it can promote AMR, increase healthcare costs, cause adverse drug reactions, and disrupt the host microbiome.

AI has emerged as a promising strategy for accelerating susceptibility prediction through the analysis of routinely generated microbiological, proteomic, genomic, and clinical data. Current applications can be broadly divided into three areas: MALDI-TOF MS-based resistance prediction, whole-genome sequencing (WGS)-based susceptibility prediction, and patient-level clinical prediction models designed to guide empirical antibiotic selection.

### 4.1. Machine-Learning Analysis of MALDI-TOF Mass Spectra for Antimicrobial-Resistance Prediction

MALDI-TOF mass spectrometry is not itself an AI technology. Conventional MALDI-TOF systems identify microorganisms by matching acquired spectra against reference databases, while other laboratory approaches may detect resistance through predefined spectral or growth-based criteria. In this review, a MALDI-TOF application was classified as AI-based only when machine-learning or deep-learning algorithms were used to identify patterns within spectral data and generate predictions of organism identity or antimicrobial resistance. Conventional MALDI-TOF assays were not included as AI evidence and are mentioned only to describe the laboratory context or provide a comparator for machine-learning-based spectral analysis.

One of the most clinically attractive applications of AI is the extraction of antimicrobial susceptibility information directly from routinely generated MALDI-TOF MS data. Conventional MALDI-TOF workflows primarily use spectral peak matching for species identification. In contrast, machine-learning algorithms can analyze the complete spectral profile and identify subtle proteomic patterns associated with antimicrobial resistance.

Weis et al. [20] trained calibrated machine-learning classifiers using more than 300,000 mass spectra linked to over 750,000 susceptibility phenotypes collected at four medical centers. For clinically relevant pathogens, the models achieved AUROCs of 0.80 for *S. aureus*, 0.74 for *Escherichia coli*, and 0.74 for *Klebsiella pneumoniae*. In a retrospective evaluation of 63 patients, AI-supported resistance prediction would have altered antimicrobial management in nine cases and would have been beneficial in eight of them (89%). These results suggest that routinely generated MALDI-TOF spectra may provide clinically useful susceptibility information before conventional phenotypic AST results become available.

More recently, Wang et al. [19] developed ANTIBIOTIC, a large-scale deep-learning platform trained using the DRIAMS database and 89,026 MALDI-TOF spectra collected during routine clinical practice. The system combined 26 LightGBM models for bacterial identification with 248 temporal convolutional network models for antimicrobial-resistance prediction. Species identification demonstrated excellent performance, with median AUROCs of 0.99 during internal validation and 0.96 during temporally independent external validation. Resistance prediction also appeared strong during internal validation, with a median AUROC of 0.94. However, the median AUROC declined to 0.55 during temporally independent external validation. Retraining with more recent local data produced only a modest improvement, increasing the median AUROC to 0.61.

Wiesmann et al. [21] evaluated six machine-learning methods using MALDI-TOF spectra from *E. coli*, *K. pneumoniae*, and *S. aureus*. The best-performing models achieved AUROCs of 0.85 for oxacillin resistance in *S. aureus*, 0.83 for ciprofloxacin resistance in *E. coli*, and 0.81 for piperacillin–tazobactam resistance in *K. pneumoniae*. Nevertheless, predictive performance declined substantially over an 18-month period, with AUROC reductions ranging from 0.10 to 0.25.

Considered together, these studies show that temporal and geographic performance degradation can substantially affect machine-learning models based on MALDI-TOF spectra. Changes in circulating bacterial clones, resistance mechanisms, antimicrobial use, patient case mix, specimen processing, laboratory equipment, and clinical practice may alter the relationship between spectral inputs and resistance phenotypes. However, the evidence presented here is derived from a limited number of MALDI-TOF studies and should not be interpreted as establishing the magnitude or frequency of model drift across all AI applications in AMR. Rather, these findings provide an important warning that performance may deteriorate after implementation and that external and temporal validation should be incorporated into future evaluations.

Performance measured during model development should therefore not be assumed to remain stable after deployment. Clinical implementation would require local external validation, continuous monitoring, periodic recalibration or retraining, and predefined thresholds for warning users or suspending a model when its performance becomes inadequate. The modest recovery observed after retraining in the study by Wang et al. further indicates that retraining alone may be insufficient when the underlying pathogen population, resistance mechanisms, or data-generating processes have changed substantially.

These findings also illustrate why AUROC alone is insufficient to determine clinical usefulness. A model with apparently good discrimination may be poorly calibrated, produce excessive false-positive predictions at the threshold used for treatment, or fail to identify a sufficiently high proportion of resistant infections to support safe prescribing. Conversely, a moderate change in AUROC may have important clinical consequences if it alters sensitivity, specificity, or predictive values at a decision threshold used to select empirical treatment.

The potential value of an earlier resistance prediction lies in shortening the time to effective therapy, reducing mismatched empirical treatment, and limiting unnecessary broad-spectrum exposure. However, most available MALDI-TOF studies evaluated technical performance retrospectively and did not prospectively demonstrate these clinical effects. Claims that a model could alter antimicrobial management were generally based on applying model outputs to historical cases or simulated treatment decisions rather than observing prescribing behavior after implementation. Accordingly, these findings indicate potential clinical utility but do not establish that AI-assisted MALDI-TOF interpretation improves antimicrobial use or patient outcomes in routine practice.

Other MALDI-TOF and growth-based methods have also been developed to accelerate the detection of carbapenemase production and phenotypic resistance [22,23]. However, because these approaches do not incorporate a machine-learning component, they were not classified as AI-based methods in the present review.

Despite this potential, important limitations remain. MALDI-TOF-based models are vulnerable to temporal performance degradation and may have limited generalizability across geographic regions, institutions, patient populations, and laboratory workflows. Their performance may also be influenced by differences in sample preparation, instrumentation, spectral preprocessing, and local resistance epidemiology. Moreover, these models infer resistance from proteomic signatures rather than directly detecting resistance mechanisms, and predictive accuracy therefore varies among pathogen–antibiotic combinations. Limited interpretability and the scarcity of prospective interventional studies demonstrating improvements in antibiotic use or patient outcomes further constrain widespread clinical implementation.

### 4.2. Artificial Intelligence-Based Resistance Prediction from Whole-Genome Sequencing

WGS represents one of the most biologically interpretable sources of information for antimicrobial susceptibility prediction because it directly characterizes genetic determinants of resistance, including resistance genes, plasmids, mobile genetic elements, and resistance-associated mutations. Machine-learning algorithms can integrate thousands of genomic features simultaneously and identify complex associations that may be overlooked by conventional rule-based interpretation.

#### 4.2.1. Tuberculosis

The most extensively investigated application of WGS-based AI involves *Mycobacterium tuberculosis*, for which conventional phenotypic susceptibility testing may require several weeks. Studies reviewed by Sharma et al. [24] indicate that random forests, support vector machines, artificial neural networks, gradient-boosting machines, and deep-learning approaches can accurately predict resistance to both first- and second-line antitubercular agents from genomic data.

A landmark study by Yang et al. [25] used WGS data from 3601 *M. tuberculosis* isolates and demonstrated that machine-learning models significantly outperformed conventional mutation-based methods. Sensitivities reached 97% for isoniazid, rifampicin, and ethambutol; 96% for ciprofloxacin and multidrug-resistant tuberculosis; and 95–96% for moxifloxacin and ofloxacin. Particularly substantial improvements were observed for pyrazinamide and streptomycin, for which sensitivity increased by 15–24% and the AUROC improved by approximately 10% compared with rule-based approaches.

Deelder et al. [26] subsequently analyzed 16,688 *M. tuberculosis* isolates using gradient-boosted trees and classification-tree models. The models achieved AUROCs exceeding 0.96 for resistance to first-line agents, amikacin, kanamycin, ciprofloxacin, and moxifloxacin, as well as for the identification of multidrug-resistant isolates. Similarly, Pruthi et al. [27] applied tree-based ensemble learning to a global dataset of 35,777 genomes linked to phenotypic susceptibility results. The models achieved AUROCs of 88.3–96.5% for first-line drugs and 84.1–95.4% for second-line drugs. Incorporating low-frequency genomic variants improved sensitivity by approximately 25% for pyrazinamide resistance and 10% for ethionamide resistance.

These findings demonstrate that machine-learning analysis of WGS data can improve both the speed and sensitivity of resistance prediction in tuberculosis, particularly for drugs for which conventional mutation catalogues remain incomplete.

#### 4.2.2. Gram-Negative Pathogens

WGS-based machine learning has also been applied to a broad range of clinically important Gram-negative pathogens. Wan et al. [28] analyzed WGS data from 1952 *E. coli* isolates linked to susceptibility phenotypes for 10 commonly used antibiotics. Across the evaluated models, AUCs ranged from 0.669 to 0.988. Gene-based and integrated-feature models showed the most robust performance, with AUROCs of 0.894–0.979 and 0.889–0.988, respectively, and near-saturated predictive accuracy for aminoglycosides and ciprofloxacin. Feature-importance analyses identified biologically plausible resistance determinants, including *blaTEM*, *blaSHV-12*, *blaCTX-M-14*, *blaOXA*, *aac(3)-IIg*, *aac(3)-IId*, *tet(A)*, and *tetR(A)*.

Focusing on resistance to a last-line antimicrobial agent, Tian et al. [29] developed a WGS-based machine-learning framework for predicting colistin resistance in *E. coli*. The models identified genomic markers associated with resistance and achieved high accuracy, sensitivity, and specificity in both training and testing datasets.

Comparable findings have been reported for *K. pneumoniae*. Zhou et al. [30] developed genomic prediction models using 4170 genomes and reported positive predictive values of 92–99% and accuracies of 95–100% in clinical specimens for susceptibility prediction to amikacin, ciprofloxacin, levofloxacin, and piperacillin–tazobactam. Sequence-based prediction reduced the time required to obtain susceptibility results by approximately 48 h compared with conventional culture-based AST.

More recently, Jia et al. [31] developed a machine-learning framework using WGS data from 5239 *K. pneumoniae* isolates collected across geographically and temporally diverse cohorts. The model predicted susceptibility to 11 antibiotics and classified binary resistant/susceptible phenotypes, resistant/intermediate/susceptible categories, and high- versus low-level resistance. AUROCs exceeded 0.90, mean categorical agreement was 96%, and very major, major, and minor error rates were 1.3%, 1.5%, and 2.4%, respectively. Predictive performance remained stable across continents, sequence types, infection sources, and collection periods, supporting the potential generalizability of this approach.

WGS-based machine-learning models have also been evaluated for other Gram-negative pathogens. In *Pseudomonas aeruginosa*, Noman et al. [32] used genomic data to predict resistance to 12 antibiotic classes and reported classification accuracies of at least 96% with random-forest models and at least 98% with BioWeka models. Accuracies reached 96.6% for meropenem, 97.3% for gentamicin, and 99.0% for ciprofloxacin resistance. Greenberg et al. [33] developed the VAMPr framework using genomic variants from clinical isolates and reported accuracies ranging from 75.6% to 98.1% across multiple pathogen–antibiotic combinations, including 98.1% for amikacin resistance.

In *Acinetobacter baumannii*, Gao et al. [34] applied random-forest, support-vector-machine, and XGBoost algorithms to WGS-derived k-mer features from 339 isolates. Categorical agreement exceeded 95%, and independent validation produced an accuracy of 96% across 13 antimicrobials. Similarly, Zheng et al. [35] evaluated six machine-learning algorithms using 1088 isolates and subsequently validated the models in an independent cohort of 508 isolates. The best-performing XGBoost model achieved a weighted accuracy of 94.4% while identifying both established carbapenemases and previously underrecognized resistance-associated determinants.

For *Salmonella enterica*, Cooper et al. [36] reported that WGS-based resistance prediction achieved an accuracy of at least 99% for most antimicrobial classes. Neuert et al. [37] similarly observed concordance exceeding 99.8% between genomic predictions and phenotypic susceptibility results across more than 52,000 isolate–antimicrobial comparisons.

Collectively, these studies suggest that AI-assisted genomic analysis can generate highly accurate and biologically interpretable susceptibility predictions across a wide range of clinically important Gram-negative pathogens.

#### 4.2.3. Gram-Positive Pathogens

Among Gram-positive pathogens, WGS-based machine-learning approaches have shown particularly promising results for *S. aureus*. Wang et al. [38] demonstrated the feasibility of predicting antimicrobial susceptibility directly from genomic data using k-mer-based machine-learning models trained on 466 isolates. Essential and categorical agreement exceeded 85% and 90%, respectively, for most antibiotics. Prediction of cefoxitin resistance also accurately identified MRSA, illustrating the potential of genomic machine learning to infer resistance phenotypes without requiring prior specification of known resistance genes.

More recently, Liu et al. [39] analyzed WGS data from 3979 *S. aureus* isolates and developed models incorporating gene-based, single-nucleotide polymorphism, k-mer-based, and integrated genomic features to predict resistance to 10 commonly used antibiotics. Overall AUROCs ranged from 0.8345 to 0.9995. Gene-based and integrated-feature models performed best, with AUROCs of 0.9311–0.9992 and 0.9313–0.9995, respectively, and outperformed SNP-only and k-mer-only models. AUROCs exceeded 0.99 for cefoxitin, methicillin, tetracycline, gentamicin, erythromycin, and clindamycin resistance, while nine of the 10 antibiotic-specific models achieved AUROCs above 0.96. Machine-learning analysis also identified previously underrecognized genomic markers associated with resistance, suggesting that these approaches may reveal predictive determinants beyond those included in conventional rule-based interpretation systems.

Overall, current evidence indicates that AI-assisted genomic analysis can provide accurate and biologically interpretable predictions of antimicrobial susceptibility and may become an important component of precision antimicrobial therapy. Nevertheless, model performance depends heavily on the size, quality, and representativeness of the training data. Accuracy may decrease when models are applied to geographically distinct bacterial populations or when new resistance mechanisms emerge. Heterogeneity in study design, genomic feature selection, resistance definitions, sequencing methods, and validation procedures also limits direct comparisons among studies. Broader clinical adoption will therefore require standardized analytical pipelines, multicenter external validation, improved model explainability, and prospective studies demonstrating measurable improvements in antibiotic selection, patient outcomes, and antimicrobial stewardship indicators.

### 4.3. Artificial Intelligence-Based Clinical Prediction Models for Empirical Antibiotic Selection

Antimicrobial resistance can also be predicted using patient-level clinical information available at the time of presentation. Rather than relying directly on microbiological, proteomic, or genomic data, these models estimate the probability of resistance from variables such as previous culture results, prior antibiotic exposure, hospitalization history, comorbidities, demographic characteristics, healthcare utilization, and local resistance epidemiology.

Models based on electronic health record (EHR) data represent a complementary approach because they can generate individualized resistance estimates immediately at presentation, before microbiological results become available. Lewin-Epstein et al. [40] applied machine-learning algorithms to electronic medical records from hospitalized patients to predict resistance to ceftazidime, gentamicin, imipenem, ofloxacin, and trimethoprim–sulfamethoxazole. Using between 2235 and 4360 susceptibility tests for each antibiotic, ensemble models achieved AUROCs of 0.73–0.79 when pathogen identity was unknown and 0.80–0.88 when bacterial species information was included. Previous resistant isolates, prior antibiotic exposure, and healthcare-associated risk factors were among the most influential predictors, demonstrating that routinely collected clinical data can support individualized empirical treatment decisions.

Feretzakis et al. [41] evaluated automated machine-learning models using 11,496 antimicrobial susceptibility records from 499 hospitalized patients. The models relied exclusively on readily available variables, including age, sex, specimen type, Gram-stain findings, and previous susceptibility results. The best-performing stacked ensemble achieved weighted AUROCs of 0.822 in the original dataset and 0.850 after class balancing. These findings support the feasibility of AI-assisted susceptibility prediction in hospitals without access to advanced genomic or proteomic technologies.

Particularly relevant evidence was provided by Yelin et al. [42], who analyzed more than 700,000 community-acquired urinary tract infections linked to over five million antibiotic-prescription records. By integrating demographic characteristics, previous urine-culture findings, and longitudinal antibiotic-exposure histories, the authors developed personalized models capable of predicting resistance to specific antibiotics before culture results became available. In retrospective analyses, these models substantially reduced the frequency of mismatched empirical treatment compared with standard prescribing practices.

EHR-based models have also been developed to predict colonization or infection with multidrug-resistant organisms at hospital admission or emergency department presentation. Target phenotypes have included carbapenem-resistant organisms, carbapenemase-producing organisms, ESBL-producing *Enterobacterales*, carbapenem-resistant Gram-negative bacteria, and MRSA. Goodman et al. [43] used more than 125 demographic and clinical variables extracted from EHRs to predict carriage of carbapenem-resistant and carbapenemase-producing organisms at admission to intensive care and transplant units. Although overall discrimination was modest, with AUROCs of 0.57–0.58, the study demonstrated the feasibility of using routinely collected data to identify patients at increased risk of carrying resistant pathogens. It also highlighted the particular difficulty of predicting low-prevalence resistance phenotypes.

Across studies, the most consistently informative predictors include recent hospitalization, prior colonization or infection with resistant organisms, previous antimicrobial exposure, comorbidity burden, healthcare utilization, and local resistance patterns. By integrating these variables, AI models may generate clinically useful resistance estimates before microbiological confirmation, support individualized empirical antibiotic selection, and reduce unnecessary broad-spectrum antibiotic exposure.

However, EHR-based models also have important limitations. Their performance depends on the completeness, accuracy, and standardization of routinely collected clinical data and may decline when they are transferred to populations with different resistance epidemiology, prescribing practices, or healthcare structures. Key predictors, including previous colonization, antibiotic exposure, and microbiological history, may be unavailable or incompletely recorded. Because these models infer resistance from clinical associations rather than directly detecting resistance mechanisms, they are vulnerable to changes in local epidemiology and the emergence of novel phenotypes. Most studies are retrospective, and resistance patterns vary substantially across institutions and over time. Consequently, the clinical utility of EHR-based prediction models depends on robust local data, external validation, regular recalibration, and continuous performance monitoring.

The clinical interpretation of antimicrobial-susceptibility models was limited by inconsistent reporting. Many studies emphasized AUROC or overall accuracy without providing outcome prevalence, calibration, decision-threshold performance, categorical agreement, or rates of very major, major, and minor errors. Overall accuracy may be misleading when resistant isolates are uncommon, because a model can achieve high accuracy by predominantly predicting the more frequent susceptible category. Moreover, very major errors are clinically more consequential than many other classification errors because they may result in an ineffective antibiotic being reported or selected. Future studies should therefore report categorical agreement and error rates using recognized susceptibility-testing definitions, in addition to discrimination, calibration, predictive values, and performance at prespecified clinical thresholds.

The main AI-based approaches to antimicrobial susceptibility prediction, together with their reported performance, potential advantages, and current limitations, are summarized in Table 2.

Only a minority of the reviewed studies connected predictive performance with a potential change in clinical management. In the retrospective evaluation by Weis et al. [20], AI-supported resistance predictions would have altered antimicrobial management in nine of 63 patients and were considered potentially beneficial in eight of these nine cases. Yelin et al. [42] reported that personalized resistance predictions could reduce mismatched empirical treatment compared with standard prescribing.

These findings are more directly relevant to clinical decision-making than AUROC alone. However, they remain retrospective or simulated assessments of what might have occurred if the models had been used. They do not establish that prospective implementation changes clinician behavior or reduces treatment failure, mortality, length of hospitalization, antimicrobial exposure, or the emergence of resistance. Prospective studies should therefore evaluate both the recommendations generated by the model and the downstream clinical consequences of acting on those recommendations.

## 5. Artificial Intelligence and Adjacent Model-Informed Approaches for Antibiotic-Dose Optimization

This section includes both AI-based dosing methods and adjacent conventional pharmacometric approaches because they address the same clinical objective; however, their methodological bases and levels of evidence are distinguished throughout the discussion.

Optimization of antibiotic dosing is a critical, although sometimes underemphasized, component of antimicrobial stewardship. Even when the appropriate agent has been selected and antimicrobial susceptibility is known, substantial interindividual pharmacokinetic variability may lead to inadequate drug exposure. Subtherapeutic concentrations can increase the risk of treatment failure and selection of resistant subpopulations, whereas excessive exposure may result in toxicity. These challenges are particularly relevant for antibiotics with narrow therapeutic windows, such as vancomycin and aminoglycosides, and in critically ill patients, whose pharmacokinetic profiles may change rapidly because of sepsis, organ dysfunction, fluid shifts, augmented renal clearance, or renal replacement therapy.

Computational approaches to precision dosing include conventional population pharmacokinetic models, Bayesian forecasting, nonparametric modeling, model averaging, digital twins, and machine-learning algorithms. These methods are not methodologically equivalent. In this review, a dosing approach was classified as AI-based only when machine learning or deep learning contributed directly to exposure prediction, pharmacokinetic-model selection or combination, dose recommendation, or treatment-course optimization. Conventional Bayesian forecasting and model-informed precision dosing without a learning component were classified as adjacent computational methods rather than AI. They are discussed separately because they provide clinically relevant comparators and illustrate the broader context in which AI-assisted dosing systems are being developed.

The most extensively studied application of AI-guided dosing involves vancomycin. Wang et al. [44] developed machine-learning models for initial and subsequent vancomycin dose recommendations using data from 2282 patients and 7912 administration records. Only 34.1% of doses prescribed in routine clinical practice achieved the target trough concentration of 14–20 μg/mL, underscoring the limitations of conventional dosing strategies. Using demographic, clinical, laboratory, and TDM variables, the proposed models produced lower prediction errors than traditional pharmacokinetic algorithms and previously published machine-learning approaches. The greatest benefit was observed in individualized dose selection, with more accurate estimation of the dose required to achieve therapeutic concentrations while reducing the risk of underexposure and excessive exposure.

Chen et al. [45] subsequently compared conventional population pharmacokinetic (PPK) models, Bayesian estimation, machine learning, and a hybrid PPK–machine-learning approach in 4059 patients with sepsis receiving vancomycin. Before drug-concentration measurements became available, the hybrid model improved exposure prediction substantially, reducing the mean absolute percentage error by 58% compared with conventional PPK models and by 17% compared with Bayesian methods. Once TDM data were available, however, Bayesian estimation remained superior, with a mean absolute percentage error of 13.4%, compared with 68.2% for conventional PPK models, 34.2% for random-forest models, and 28.5% for hybrid models. These findings suggest that AI may be particularly valuable during the initial phase of therapy, when measured drug concentrations are unavailable and conventional precision-dosing approaches are less reliable.

A different strategy was evaluated by van Os et al. [46], who analyzed 343,636 vancomycin TDM records from 156 healthcare centers. Rather than directly predicting the optimal dose, the investigators developed an XGBoost-based framework to identify which of six population pharmacokinetic models was most appropriate for each patient. The system ranked and combined candidate models according to patient-specific clinical characteristics and then generated individualized exposure estimates and dosing recommendations. Compared with individual pharmacokinetic models, body mass index-based model selection, and naïve averaging, machine-learning-guided model selection increased the proportion of predicted concentrations within 80–125% of observed values and reduced prediction bias. This approach illustrates how AI may improve precision dosing by selecting or combining the most appropriate pharmacokinetic model for a given patient instead of applying a single population model universally.

Although vancomycin remains the most extensively investigated antibiotic in this field, AI-supported and model-informed approaches are also being explored for β-lactams and aminoglycosides. In critically ill patients receiving meropenem, precision-dosing frameworks have been developed to address the marked pharmacokinetic variability associated with sepsis and continuous renal replacement therapy (CRRT). Schatz et al. [47] showed that incorporating TDM data into Bayesian dosing models reduced the median absolute prediction error from 35.4% to 25.0% and the median prediction error from 21.8% to 4.6% in critically ill patients undergoing CRRT. Machine-learning models designed specifically for meropenem dosing during CRRT have likewise shown promising performance for individualized dose optimization [48].

Comparable approaches have been investigated for piperacillin–tazobactam. Haefliger et al. [49] reported that model-informed precision dosing increased the probability of achieving target piperacillin exposure from 55% with empirical TDM to 83–94% when Bayesian model-informed dose adjustments were applied. In a multicenter study of 561 critically ill patients and 3654 TDM samples, Schatz et al. [50] found that a model-averaging algorithm achieved target attainment rates above 77% after a single concentration measurement and above 90% after incorporation of a second sample obtained within 24 h.

Similar progress has been reported for cefepime. Alshaer et al. [51] developed and validated a precision-dosing platform using data from 680 patients. The system demonstrated excellent predictive performance, with an R^2^ greater than 0.98 and a median concentration-prediction bias of only 4%, enabling individualized dose recommendations based on renal function and pharmacodynamic targets.

Aminoglycosides represent another promising area for AI-assisted precision dosing. Prunella et al. [52] developed an evolutionary digital-twin framework for amikacin dosing in neonatal sepsis. The platform integrated physiologically based pharmacokinetics, bacterial population dynamics, resistance evolution, and a long short-term memory neural network trained on renal-function trajectories. Calibrated using data from 1634 neonates, the framework enabled in silico optimization of complete treatment courses while balancing antimicrobial efficacy, toxicity, and suppression of resistance emergence.

Despite these advances, evidence supporting AI-assisted and model-informed antibiotic dosing remains limited. Most studies have focused on vancomycin, whereas evidence for other antimicrobials is less mature. Many investigations are retrospective or evaluate pharmacokinetic prediction rather than clinical outcomes, and external validation across institutions, patient populations, and healthcare systems remains incomplete.

Several studies reported clinically proximal outcomes rather than AUROC. For example, model-informed piperacillin–tazobactam dosing increased predicted target attainment from 55% with empirical therapeutic drug monitoring to 83–94% after Bayesian dose adjustment [49]. In a multicenter study, model averaging produced target-attainment rates above 77% after one concentration measurement and above 90% after incorporation of a second measurement obtained within 24 h [50]. These outcomes are more clinically interpretable than discrimination alone, but pharmacokinetic target attainment remains an intermediate outcome and should not be equated with demonstrated clinical benefit.

Most reported benefits of AI-assisted or model-informed dosing were based on retrospective pharmacokinetic analyses, prediction-error comparisons, simulations, or estimated target attainment. These outcomes demonstrate technical performance or potential dosing utility but do not establish that implementation reduces nephrotoxicity, other adverse events, treatment failure, mortality, length of hospitalization, or selection of resistant organisms. Pharmacokinetic target attainment is an important intermediate outcome, but it should not be treated as equivalent to demonstrated clinical benefit.

The clinical value of dosing technologies cannot be inferred from concentration-prediction accuracy or target attainment alone. Prospective trials must determine whether model-informed dosing reduces toxicity, treatment failure, mortality, length of hospitalization, antimicrobial consumption, or the emergence of resistance. This evidence gap has prompted randomized studies such as VANC-DOS, which was designed to determine whether model-informed vancomycin dosing improves pharmacokinetic target attainment and reduces acute kidney injury compared with standard care [53]. However, the cited publication describes the study design, and no clinical benefit was attributed to the intervention in the absence of publicly available trial results.

Representative AI-assisted and model-informed approaches to antibiotic dose optimization, along with their principal findings and potential clinical applications, are summarized in Table 3.

## 6. Artificial Intelligence and the Development of New Antibiotics

Over the past three decades, antibiotic innovation has slowed substantially, while AMR has continued to increase worldwide. During the so-called “golden era” of antibiotic discovery, from the 1950s to the 1980s, more than 20 antibiotic classes entered clinical practice, including aminoglycosides, cephalosporins, glycopeptides, and carbapenems. These classes encompassed numerous highly effective agents that transformed the treatment of bacterial infections. Since the 1980s, however, the continued emergence and spread of multidrug-resistant pathogens have been accompanied by the introduction of only a limited number of genuinely novel antibiotic classes, generally represented by relatively few clinically relevant compounds.

Although agents such as the oxazolidinone linezolid, the lipopeptide daptomycin, and the siderophore cephalosporin cefiderocol have expanded the available therapeutic armamentarium, these advances have been insufficient to offset the rapid evolution of AMR. A widening gap has therefore emerged between the pace at which resistance develops and the rate at which new antimicrobial agents become available, creating a major vulnerability in the global response to drug-resistant infections [54,55].

This innovation deficit reflects the intrinsic scientific, clinical, and economic challenges of antibiotic research and development. Traditional discovery programs are costly, time-consuming, and characterized by exceptionally high attrition rates, often requiring the screening of thousands of compounds to identify a single candidate suitable for further development. Additional barriers include the difficulty of identifying novel bacterial targets, the rapid emergence of resistance to newly introduced agents, rising development costs, lengthy regulatory pathways, and limited commercial returns. Together, these factors have contributed to the contraction of the antibacterial pipeline.

Against this background, AI has emerged as a promising tool for revitalizing antimicrobial discovery. AI-based approaches can accelerate target identification, virtual screening, candidate prioritization, molecular optimization, and de novo compound design. By analyzing large chemical and biological datasets, these systems may identify structurally novel antibacterial compounds and previously unexplored chemical scaffolds that are difficult to detect using conventional medicinal chemistry methods [7,56,57].

AI has also enabled the *de novo* design of antimicrobial molecules and peptides, thereby expanding the searchable chemical space beyond compounds already represented in existing antibiotic libraries [7,57]. One of the most prominent examples is halicin, which was identified through deep-learning screening of large chemical libraries. Halicin demonstrated activity against several multidrug-resistant pathogens, including carbapenem-resistant *Enterobacterales*, *A. baumannii*, and *M. tuberculosis*. Its antibacterial activity was associated with disruption of the bacterial transmembrane electrochemical gradient, representing a mechanism distinct from those of many conventional antibiotics [56].

Another important example is abaucin, which was identified using machine-learning algorithms trained specifically to detect compounds active against *A. baumannii*. Abaucin showed selective activity against multidrug-resistant *A. baumannii* strains and demonstrated efficacy in murine infection models [58,59]. This pathogen-specific approach illustrates how AI may be used not only to identify broad-spectrum compounds but also to design or prioritize narrow-spectrum agents that target clinically important resistant organisms while potentially limiting disruption of the commensal microbiota.

Beyond small molecules, AI-driven generative models have been applied to the discovery and design of novel antimicrobial peptides (AMPs). These approaches have generated candidates with predicted or experimentally confirmed activity against MRSA, vancomycin-resistant enterococci, multidrug-resistant *P. aeruginosa*, and carbapenem-resistant Gram-negative bacteria [60]. Porto et al. [61], for example, used computational and machine-learning-based methods to optimize synthetic AMPs with broad-spectrum antibacterial activity and favorable predicted toxicity profiles, several of which were subsequently validated experimentally. Similarly, Capecchi and Reymond [62] applied machine-learning-guided peptide design to identify novel AMP candidates with activity against clinically relevant resistant pathogens.

Collectively, these studies demonstrate that AI can extend beyond conventional compound screening and contribute directly to the generation and prioritization of previously unexplored antimicrobial structures. In principle, this capability could help identify compounds with novel mechanisms of action, overcome existing resistance mechanisms, and expand the early-stage antibiotic pipeline.

Nevertheless, the practical impact of AI on antibiotic development remains limited. Most reported successes are still confined to discovery and preclinical research, and very few AI-identified candidates have progressed to advanced clinical development. To date, no AI-discovered antibiotic has substantially altered the global AMR landscape. One major limitation is the dependence of AI models on relatively small, heterogeneous, incomplete, or biased chemical and microbiological datasets, which may reduce robustness and generalizability. Models may also preferentially identify compounds that resemble known antibiotics rather than truly novel scaffolds capable of overcoming future resistance mechanisms.

Moreover, promising in silico activity frequently fails to translate into successful drug development. Antibacterial potency alone does not ensure acceptable pharmacokinetics, safety, tissue penetration, stability, manufacturability, or clinical efficacy. Current AI systems also have limited capacity to reproduce the complexity of host–pathogen interactions, microbial ecology, immune responses, and the evolutionary dynamics of resistance. Experimental validation, medicinal chemistry optimization, toxicological assessment, and clinical testing therefore remain indispensable.

Finally, AI cannot by itself resolve the economic and structural barriers that continue to constrain antibiotic innovation, including high development costs, lengthy regulatory processes, restricted use of newly approved agents, and poor commercial returns. AI should therefore be regarded as an enabling technology that can improve the efficiency and scope of early-stage discovery, rather than as a standalone solution to the antibiotic-development crisis.

The principal applications of AI across the antibiotic discovery and development pathway, including representative examples, potential contributions, and current limitations, are summarized in Table 4.

Performance assessment in antibiotic discovery also differs from clinical prediction. AUROC or compound-ranking accuracy may indicate that a model can enrich a screening library for active molecules, but these measures do not demonstrate that the selected compounds have acceptable pharmacokinetics, toxicity, tissue penetration, stability, manufacturability, resistance barriers, or clinical efficacy. The most meaningful outcomes therefore include experimentally confirmed hit rates, structural novelty, activity against resistant clinical isolates, in vivo efficacy, toxicological findings, progression through medicinal-chemistry optimization, and advancement into clinical development.

Most AI-identified antimicrobial candidates remain at the in vitro or preclinical stage. Consequently, current evidence supports the ability of AI to prioritize or generate promising candidates but does not demonstrate that it reduces development costs, shortens the overall development timeline, or increases the probability of regulatory approval. The absence of prospective interventional trials in the antibiotic-discovery domain further illustrates the distance between computational performance and clinical translation.

## 7. Prospective Interventional Evidence

Within the prespecified registry search, five prospective interventional studies met the operational definition of an AI-based intervention [63,64,65,66,67]. Two concerned AI-assisted diagnosis or antibiotic-use decisions, and three concerned resistance prediction or antimicrobial-stewardship decision support. One additional randomized study, VANC-DOS, evaluated conventional model-informed precision dosing of vancomycin but did not include a clearly identified machine-learning component [68]. It was therefore considered separately as adjacent pharmacometric evidence rather than counted as an AI intervention. No eligible prospective interventional study of AI-assisted antibiotic discovery was identified within the registries and search strategy used.

Thus, despite the large body of retrospective model-development and validation research, only six prospective interventional studies were identified, and prospective evidence was absent from the antibiotic-discovery domain. This marked imbalance represents a principal finding of the review. Furthermore, registration as an interventional study does not demonstrate clinical benefit: several studies were ongoing or lacked publicly available results at the search date. The available registry evidence therefore supports the conclusion that prospective evaluation remains at an early stage.

### Economic and Implementation Considerations

The economic implications of AI in AMR remain insufficiently studied. Potential savings may result from faster diagnosis, earlier appropriate treatment, reduced use of unnecessary or broad-spectrum antibiotics, fewer adverse drug events, shorter hospitalization, more efficient therapeutic drug monitoring, and prioritization of compounds before expensive experimental screening. AI-assisted discovery may also reduce the number of inactive compounds advanced to laboratory testing. These potential benefits cannot, however, be assumed from improvements in predictive accuracy.

Implementation entails costs associated with data acquisition and curation, computing infrastructure, software development or licensing, integration with laboratory information systems and electronic prescribing platforms, cybersecurity, regulatory compliance, external validation, technical support, performance monitoring, recalibration, and staff training. Model drift may further increase expenditure by requiring repeated validation and retraining. Institutions with limited digital infrastructure may face particularly high implementation costs, potentially increasing inequities between healthcare systems.

Future prospective studies should therefore include economic outcomes from the beginning of their design. Relevant measures include total implementation and maintenance costs, budget impact, cost per inappropriate prescription avoided, cost per additional patient receiving appropriate initial therapy, cost per adverse event prevented, cost per quality-adjusted life-year gained, and the opportunity costs associated with false-positive and false-negative recommendations. For AI-assisted antibiotic discovery, evaluations should examine whether computational prioritization reduces the cost or duration of screening and whether this advantage persists through preclinical and clinical development. Formal cost-effectiveness evidence will be necessary to determine whether technical improvements justify widespread implementation.

## 8. Conclusions

AI has demonstrated promising technical performance across antimicrobial stewardship, infection diagnosis, resistance prediction, precision dosing, and antibiotic discovery. Several systems achieve high internal discrimination, identify clinically relevant patterns in complex datasets, or improve pharmacokinetic predictions. These positive findings establish the potential of AI to support earlier and more individualized decision-making.

Nevertheless, the evidence reviewed here identifies a substantial translational gap. High internal performance may not persist across institutions or periods. The substantial decline in resistance-prediction performance reported in two MALDI-TOF studies illustrates the potential importance of temporal and geographic model drift. Although this limited evidence cannot be generalized quantitatively to all AI applications in AMR, it supports the need for local external validation, continuous performance monitoring, periodic recalibration, and predefined procedures for reviewing or suspending models whose performance becomes inadequate.

The clinical meaning of the reported performance measures also remains uncertain. AUROC measures discrimination but does not establish calibration, usefulness at a particular decision threshold, effects on prescribing, patient benefit, or economic value. Although some studies reported clinically proximal outcomes, such as changes in antimicrobial selection, reductions in mismatched treatment, or improved pharmacokinetic target attainment, relatively few assessed treatment failure, toxicity, mortality, length of hospitalization, antimicrobial consumption, emergence of resistance, or cost-effectiveness.

The registry search reinforces this conclusion. The structured registry search identified five prospective interventional studies of AI-based applications and one additional randomized study of conventional model-informed precision dosing. This scarcity contrasts with the much larger number of retrospective model-development studies and indicates that prospective clinical evaluation remains at an early stage.

AI should therefore currently be regarded as a potentially valuable complement to, rather than a replacement for, established diagnostic, antimicrobial-stewardship, surveillance, precision-dosing, and drug-development strategies. Future research should move beyond isolated measures of technical performance and conduct prospective, multicenter, and pragmatic evaluations of clinically meaningful outcomes. Such studies should incorporate calibration, decision-threshold analysis, continuous monitoring for model drift, implementation feasibility, clinician behavior, patient safety, equity, budget impact, and cost-effectiveness. The ultimate value of AI will depend not on whether it can generate accurate predictions under development conditions, but on whether those predictions remain reliable and can be translated into safer prescribing, better patient outcomes, reduced antimicrobial exposure, and measurable reductions in AMR.

## Figures and Tables

**Table 1:** Main applications of artificial intelligence in infectious-disease diagnosis and antimicrobial stewardship.

Clinical Application	Data Source or Approach	Representative Evidence	Main Findings	Potential Contribution to Antimicrobial Stewardship
Differentiation of bacterial and viral infections	Routine laboratory variables, demographic data, biomarkers, and machine-learning classification	Gunčar et al. [11]	Model incorporating 16 laboratory parameters, CRP, age, and sex achieved an AUROC of 0.905, with 82.2% accuracy, 79.7% sensitivity, and 84.5% specificity.	May reduce unnecessary antibiotic prescribing, particularly in patients with intermediate biomarker values.
Host-response classification	Host mRNA expression and neural-network analysis	Mayhew et al. [12]	A 29-mRNA classifier achieved AUROCs of 0.92 for bacterial-versus-other and viral-versus-other classifications.	Provides etiologic information when microbiological confirmation is unavailable or delayed.
Multiclass infection diagnosis	Integrated transcriptomic data and pretrained neural networks	Li et al. [13]	The bvnGPS model achieved AUROCs of 0.953 and 0.956 for bacterial and viral infections; external-validation AUROCs were 0.988 and 0.994.	Supports individualized classification of bacterial, viral, and noninfectious conditions.
Gene-expression-based diagnosis	Ensemble machine learning using selected host genes	Shen et al. [14]	InfectDiagno achieved an AUROC of 0.95 and correctly classified approximately 95% of samples in prospective validation.	May improve diagnostic confidence and reduce empirical antibiotic use.
Prediction of bacteremia	Clinical variables, PCT, and machine-learning risk models	Kaal et al. [15]	External-validation AUROC was 0.87; estimated blood-culture use was reduced by 29% while missing 1.1% of bacteremia cases.	May improve diagnostic stewardship and reduce unnecessary investigations.
Uncertainty-aware bacteremia prediction	Structured and unstructured emergency department data and Bayesian neural networks	Choi et al. [16]	External-validation AUROC was 0.738 with 92.7% sensitivity; physician performance improved when AI estimates were provided.	May support decision-making in diagnostically uncertain cases.
Early sepsis prediction	Multinational intensive-care data and deep learning	Moor et al. [17]	The model identified 80% of septic patients a median of 3.7 h before clinical onset; AUROC was 0.846.	May facilitate earlier treatment in high-risk patients.
Prediction of deterioration and antibiotic need in children	Electronic health record data and two-stage machine learning	Velez et al. [18]	Models achieved negative predictive values above 96% for clinical deterioration and bacteremia.	May identify low-risk children in whom antibiotics can be safely withheld.
AI-enhanced MALDI-TOF identification	Full-spectrum MALDI-TOF data and deep learning	Wang et al. [19]	Median AUCs for species identification were 0.99 internally and 0.96 in temporal external validation.	May accelerate pathogen identification without additional laboratory procedures.

Abbreviations: AI, artificial intelligence; AUC, area under the curve; AUROC, area under the receiver operating characteristic curve; bvnGPS, bacterial–viral–noninfected Gene Pair Signature; CRP, C-reactive protein; MALDI-TOF, matrix-assisted laser desorption/ionization time-of-flight; mRNA, messenger RNA; PCT, procalcitonin.

**Table 2:** Artificial intelligence approaches for antimicrobial susceptibility prediction.

AI Approach	Pathogens or Setting	Representative Studies	Reported Performance	Principal Advantages	Main Limitations
MALDI-TOF-based resistance prediction	*S. aureus*, *E. coli*, and *K. Pneumoniae*	Weis et al. [20]	AUROCs of 0.80, 0.74, and 0.74, respectively.	Uses routinely generated data and may provide resistance estimates before phenotypic AST.	Moderate accuracy for some pathogen–antibiotic combinations and limited prospective evidence.
Deep-learning analysis of MALDI-TOF spectra	Multiple bacterial species and antibiotic combinations	Wang et al. [19]	Resistance-prediction AUROC decreased from 0.94 internally to 0.55 during temporal external validation.	Enables simultaneous species identification and resistance prediction.	Strong susceptibility to temporal and geographic model drift.
Machine-learning analysis of MALDI-TOF spectra	*S. aureus*, *E. coli*, and *K. Pneumoniae*	Wiesmann et al. [21]	AUROCs of 0.85, 0.83, and 0.81 for selected resistance phenotypes.	May accelerate early susceptibility assessment.	AUROC declined by 0.10–0.25 over 18 months, requiring regular retraining.
WGS-based resistance prediction in tuberculosis	*M. tuberculosis*	Yang et al. [25]; Deelder et al. [26]; Pruthi et al. [27]	Sensitivities up to 97%; AUROCs generally above 0.84 and frequently above 0.96.	Especially valuable because conventional phenotypic testing may require weeks.	Dependent on genomic diversity, resistance catalogues, and sequencing infrastructure.
WGS-based prediction in *E. coli*	Multiple antibiotics, including aminoglycosides, fluoroquinolones, and colistin	Wan et al. [28]; Tian et al. [29]	AUROCs ranged from 0.669 to 0.988; gene-based models generally performed best.	Identifies biologically plausible resistance determinants.	Performance may vary with population structure and rare variants.
WGS-based prediction in *K. pneumoniae*	Multiple antibiotic classes	Zhou et al. [30]; Jia et al. [31]	PPVs of 92–99%; categorical agreement of 96%; AUROCs above 0.90.	May reduce time to susceptibility results by approximately 48 h.	Requires standardized genomic pipelines and external validation.
WGS-based prediction in other Gram-negative pathogens	*P. aeruginosa*, *A. baumannii*, and *S. enterica*	Noman et al. [32]; Greenberg et al. [33]; Gao et al. [34]; Zheng et al. [35]; Cooper et al. [36]; Neuert et al. [37]	Reported accuracies generally exceeded 94%, reaching more than 99% in selected analyses.	Supports rapid, biologically interpretable susceptibility prediction.	Heterogeneity in datasets, resistance definitions, and validation methods.
WGS-based prediction in Gram-positive pathogens	*S. aureus*	Wang et al. [38]; Liu et al. [39]	AUROCs ranged from 0.8345 to 0.9995; 9 of 10 models achieved AUROCs above 0.96.	May identify known and underrecognized genomic resistance markers.	Requires large, representative genomic datasets.
EHR-based empirical therapy prediction	Hospitalized patients and community-acquired urinary tract infections	Lewin-Epstein et al. [40]; Feretzakis et al. [41]; Yelin et al. [42]	AUROCs generally ranged from 0.73 to 0.88; retrospective analyses showed fewer mismatched prescriptions.	Provides immediate individualized estimates before laboratory results.	Dependent on data completeness, local epidemiology, and prescribing patterns.
Prediction of multidrug-resistant organism carriage	Intensive-care and transplant populations	Goodman et al. [43]	AUROCs of 0.57–0.58.	Demonstrates feasibility of admission-based risk prediction.	Limited discrimination for low-prevalence resistance phenotypes.

Abbreviations: AI, artificial intelligence; AST, antimicrobial susceptibility testing; AUROC, area under the receiver operating characteristic curve; EHR, electronic health record; MALDI-TOF, matrix-assisted laser desorption/ionization time-of-flight; PPV, positive predictive value; WGS, whole-genome sequencing.

**Table 3:** Artificial intelligence and model-informed approaches for antibiotic dose optimization.

Antibiotic or Class	AI or Modeling Strategy	Study Population	Main Findings	Potential Clinical Relevance
Vancomycin	Machine-learning models for initial and subsequent dose recommendations	2282 patients and 7912 administration records	Only 34.1% of routine doses achieved target trough concentrations; AI models reduced prediction error compared with conventional approaches [44].	May improve individualized dose selection and reduce underexposure or toxicity.
Vancomycin	Hybrid population pharmacokinetic–machine-learning model	4059 patients with sepsis	Before TDM data were available, the hybrid model reduced mean absolute percentage error by 58% versus conventional PPK models and by 17% versus Bayesian methods [45].	May be most useful during the initial phase of treatment.
Vancomycin	Machine-learning-guided selection and averaging of pharmacokinetic models	343,636 TDM records from 156 healthcare centers	Increased the proportion of predicted concentrations within 80–125% of observed values and reduced prediction bias [46].	Enables patient-specific selection of the most appropriate pharmacokinetic model.
Meropenem	Bayesian model-informed precision dosing with TDM	Critically ill patients receiving CRRT	Median absolute prediction error decreased from 35.4% to 25.0%, and median prediction error from 21.8% to 4.6% [47].	May improve target attainment in patients with highly variable pharmacokinetics.
Meropenem	Machine-learning-based individualized dosing	Patients receiving CRRT	Models showed promising predictive performance for dose optimization [48].	May support individualized dosing in complex critical-care settings.
Piperacillin–tazobactam	Bayesian model-informed precision dosing	Patients undergoing TDM	Probability of target attainment increased from 55% with empirical TDM to 83–94% with model-informed adjustment [49].	May reduce subtherapeutic exposure in critically ill patients.
Piperacillin–tazobactam	Multi-model averaging	561 critically ill patients and 3654 TDM samples	Target attainment exceeded 77% after one concentration and 90% after a second sample within 24 h [50].	Supports dynamic dose adaptation during treatment.
Cefepime	Nonparametric precision-dosing platform	680 patients	Predictive performance exceeded R^2^ = 0.98, with a median concentration-prediction bias of 4% [51].	May enable dose adjustment according to renal function and pharmacodynamic targets.
Amikacin	Evolutionary digital-twin framework integrating pharmacokinetics, bacterial dynamics, and renal trajectories	Data from 1634 neonates	Enabled in silico optimization of complete treatment courses while balancing efficacy, toxicity, and resistance suppression [52].	May support precision dosing in neonatal sepsis.
Vancomycin	Prospective randomized evaluation of model-informed dosing	Adults receiving continuous-infusion vancomycin	Randomized trial designed to evaluate pharmacokinetic target attainment and acute kidney injury; the cited publication reports the study protocol rather than demonstrated clinical outcomes [53].	May provide prospective evidence; clinical benefit cannot be inferred until trial results are available.

Abbreviations: AI, artificial intelligence; CRRT, continuous renal replacement therapy; PPK, population pharmacokinetic; TDM, therapeutic drug monitoring; VANC-DOS, model-informed vancomycin dosing trial.

**Table 4:** Artificial intelligence applications in antibiotic discovery and development.

Application	AI Strategy	Representative Example	Main Contribution	Current Limitations
Target identification	Analysis of genomic, transcriptomic, structural, and biological datasets	AI-based prioritization of bacterial targets [7,56,57]	May identify novel and biologically relevant targets more rapidly than conventional approaches.	Predictions depend on the quality and completeness of biological datasets.
Virtual compound screening	Deep-learning analysis of large chemical libraries	Halicin [56]	Identified a structurally distinct compound with activity against multidrug-resistant pathogens, including carbapenem-resistant Enterobacterales and A. baumannii.	Most evidence remains preclinical.
Pathogen-specific antibiotic discovery	Machine-learning models trained to identify activity against a selected organism	Abaucin [58,59]	Demonstrated selective activity against multidrug-resistant A. baumannii and efficacy in murine models.	Clinical pharmacokinetics, safety, and efficacy remain to be established.
De novo molecular design	Generative AI and deep-learning-based creation of novel chemical structures	Novel antimicrobial scaffolds [7,57]	Expands the searchable chemical space beyond existing antibiotic databases.	Generated compounds may resemble known structures or lack drug-like properties.
Antimicrobial peptide discovery	Machine-learning prediction, generation, mining, and optimization	AI-designed antimicrobial peptides [60]	Enables rapid identification of peptides with predicted activity against resistant bacteria.	Susceptibility to degradation, toxicity, delivery challenges, and manufacturing complexity.
Peptide optimization	Computational redesign and machine-learning-guided sequence selection	Porto et al. [61]	Produced optimized synthetic peptides with broad-spectrum activity and favorable predicted toxicity.	Experimental and clinical validation remain necessary.
Peptide library exploration	Machine-learning-guided design and screening	Capecchi and Reymond [62]	Identified novel peptide candidates active against clinically relevant resistant pathogens.	Translation from in vitro activity to clinical efficacy remains uncertain.
Candidate prioritization	Integration of activity, structure, toxicity, and physicochemical properties	AI-supported lead selection [7,56,57]	May reduce the number of compounds requiring experimental screening.	Models cannot fully predict tissue penetration, manufacturability, or human safety.
Molecular optimization	Prediction of structure–activity relationships and compound properties	Optimization of early antimicrobial leads [7,56,57]	May improve potency, selectivity, and drug-like characteristics.	In silico improvements may not translate into favorable in vivo performance.
Resistance-aware design	Modeling of bacterial evolution and potential resistance mechanisms	Emerging generative and predictive approaches [7,57]	May support development of compounds less vulnerable to existing resistance mechanisms.	Resistance evolution and host–pathogen interactions remain difficult to model accurately.

Abbreviations: AI, artificial intelligence; AMR, antimicrobial resistance.

## Data Availability

No new data were created or analyzed in this study. Data sharing is not applicable to this article.
